# ENSO-induced precipitation seasonality in the Chinese loess plateau during 1767–2050 CE

**DOI:** 10.1016/j.fmre.2025.04.010

**Published:** 2025-04-23

**Authors:** Liang Yi, Liangcheng Tan

**Affiliations:** a State Key Laboratory of Marine Geology, Tongji University, Shanghai 200092, China; b Institute of Global Environment Change, Xi’an Jiaotong University, Xi’an 710054, China; c State Key Laboratory of Loess Science, Institute of Earth Environment, Chinese Academy of Sciences, Xi’an 710061, China

**Keywords:** Magnetic scanning, Stalagmite, Precipitation seasonality, Chinese loess plateau, El Niño-southern oscillation

## Abstract

Climate change has not only altered the total amount of rainfall but also significantly impacted its seasonal distribution. This alteration is particularly critical in monsoonal regions, where the availability and timing of water resources are essential for agricultural activities and ecological sustainability. However, the understanding of precipitation seasonality was largely constrained by the limitations of observational data. In this study, we present a reconstruction of seasonal precipitation patterns derived from the magnetic properties of a stalagmite collected from the Chinese Loess Plateau, which serves as a proxy for precipitation seasonality from 1767 to CE 2006. Our results indicate that magnetic-based precipitation seasonality exhibits significant fluctuations characterized by quasiperiodic oscillations with periodicities of 2–7 and 14 years. We find an upward trend in precipitation seasonality since the 1980s, suggesting that North China may be entering a new phase of increased rainfall seasonality, and by applying a convolutional-neural-network forecasting model, this upward trend could be expected to extend till CE 2050. We then observe a strong correlation between changes in this seasonality proxy and El Niño-Southern Oscillation (ENSO) conditions, inferring that El Niño events amplify precipitation seasonality in North China on decadal timescales over the past three centuries and sustaining at least to CE 2050. Therefore, our reconstruction demonstrates the importance of considering ENSO forcing when assessing precipitation seasonality and, in turn, provides alternative ways to document ENSO variability in the past.

## Graphical abstract

Magnetic scanning of a stalagmite sample collected from Chinese Loess Plateau (CLP) assists to reconstruct precipitation seasonality in the past and shows that El Nino events amplified its variability in the past centuries and the coming decades.

**Figure:**
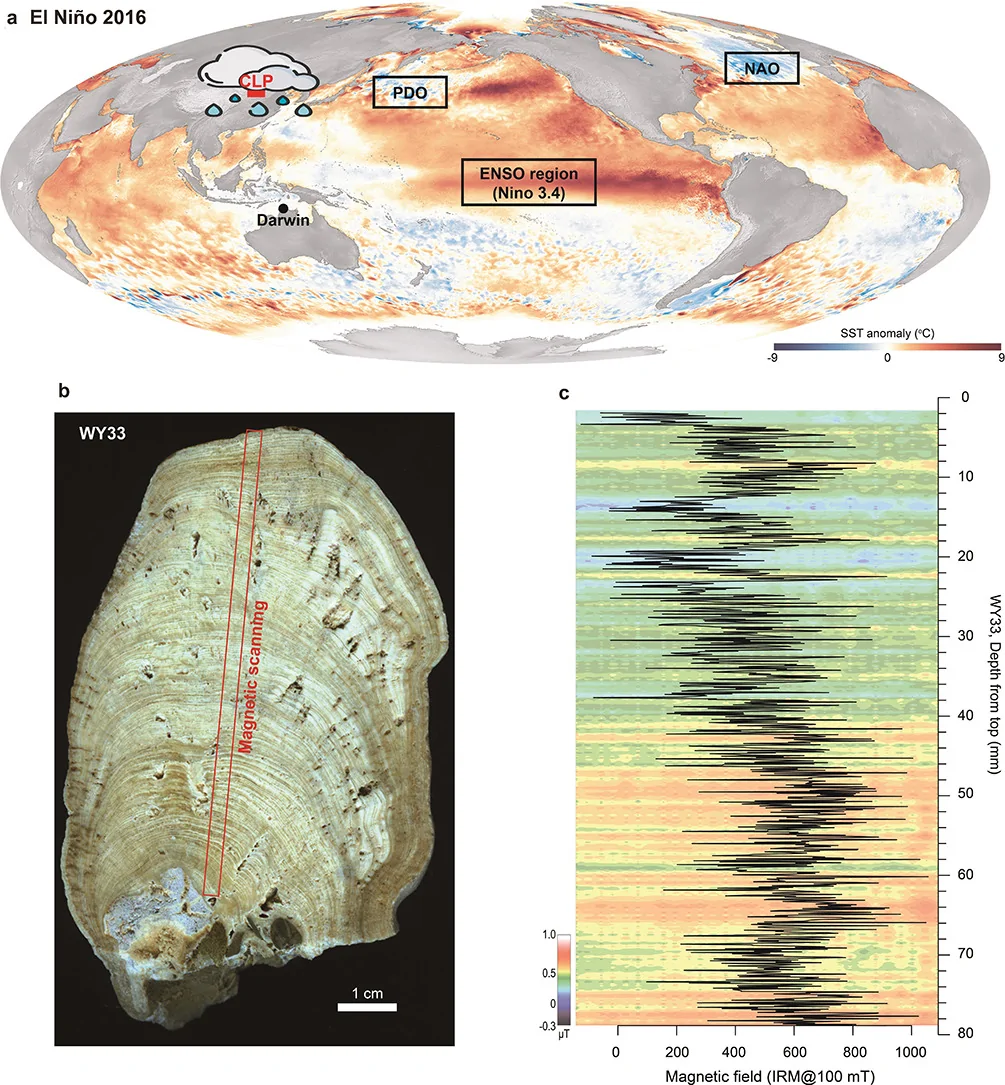

## 1 Introduction

Ecosystems in tropical and monsoonal regions are highly sensitive not only to total annual rainfall but also to its seasonal distribution, known as precipitation seasonality, which reflects intra-annual variance in precipitation [[1], [2], [3]]. In regions with high rainfall, an increase in precipitation seasonality often results in a higher contribution of torrential rainfall to the annual total, frequently leading to floods. Consequently, precipitation seasonality plays a critical role in agriculture activities [4,5], water-resource management [6,7], and ecological vulnerability [8,9].

Despite its importance, the analysis of precipitation seasonality and its response to global changes has been limited by short-term meteorological observations and the scarcity of paleoclimatic proxies with monthly or seasonal resolution. While global warming is expected to increase rainfall extremes, such as droughts and floods [[10], [11], [12]], the response of rainfall seasonality remains a subject of intense debate [13,14]. For example, some regions have experienced changes in precipitation seasonality over the past few decades [2,15], while others, like North America and East Africa, have shown no significant changes [16,17]. Furthermore, precipitation seasonality changes observed over short periods, such as from the 1960s to 2010s in China [18,19], may yield conflicting responses to global warming and El Niño-Southern Oscillation (ENSO) [14].

ENSO, the most influential global climate phenomenon [20], is known to drive interannual variability in precipitation across China, including rainfall magnitude [21], extreme rainfall events [22], and precipitation seasonality [14] over the past seventy years. While the relationship between ENSO and rainfall magnitude and extreme events has been well-documented [[23], [24], [25]], its connection to rainfall seasonality has received less attention.

To better understand the variability in precipitation seasonality over recent centuries in China, we reconstructed seasonal precipitation for the period of 1767–2006 CE, examining the relationship between precipitation seasonality and ENSO patterns through high-resolution magnetic scanning of a stalagmite (WY33) collected from Wuya Cave in the Chinese Loess Plateau (Fig. 1). Our results suggest that El Niño events may amplify precipitation seasonality in North China on decadal timescales, providing new insights into the long-term dynamics of precipitation seasonality in monsoonal regions.

**Fig. 1:**
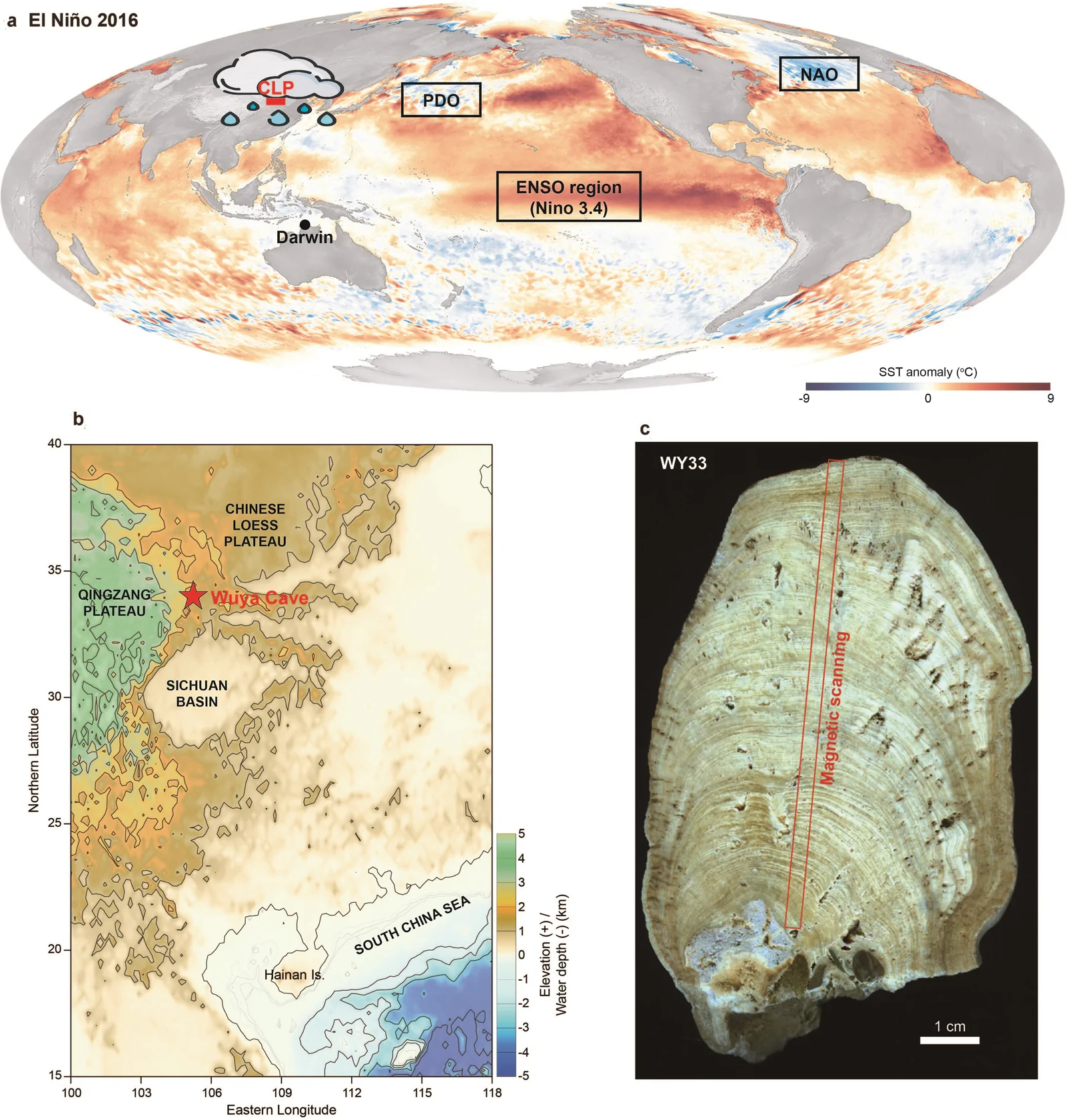
**Map showing the study area and the sample.** a, global sea surface temperature (SST) changes in 2016, indicating an El Niño event. PDO, Pacific decadal oscillation; NAO, North Atlantic Oscillation; CLP, Chinese Loess Plateau. The SST data in the Pacific were from ERSST [26]. b, Topography of the study area. c, Stalagmite WY33 collected from Wuya Cave, Chinese Loess Plateau, with a rectangle labelling the segment polished for magnetic scanning. Monthly rainfall data (1951–2004 CE) for seasonality calculation were from the China Meteorological Administration.

## 2 Methods

### 2.1 The studied stalagmite (WY33)

Wuya Cave (33.82° N, 105.43° E, 1370 m above sea level) is located in the Chinese Loess Plateau (Fig. 1). The regional climate is semiarid, with an average annual precipitation of 470 mm and an average annual temperature of 14.9 °C. Precipitation seasonality in this area is significant, with a rainfall variance of 150 mm between four seasons, which accounts for ∼30% of the annual total. According to meteorological records from the nearby Wudu station, located about 60 km southwest of Wuya Cave, the summer monsoon brings warm, humid air, resulting in substantial rainfall, with ∼80% of the annual precipitation occurring between May and September. During the winter months, the Siberian-Mongolian High and westerly winds dominate, bringing cold and dry conditions to the region [25].

Stalagmite WY33 was collected from a chamber about 500 m inside Wuya Cave. The stalagmite WY33 has a length of 84.5 mm and was actively receiving drip water at the time of collection. The polished cross-section of WY33 exhibits alternating dark, compact layers and white, porous layers (Fig. 1). Lamina counting identified 262 ± 10 layers, corresponding to a mean growth rate of 0.361 mm/year, which is comparable with ^210^Pb dating results [27]. Based on these observations, the layers are interpreted to represent annual deposition, spanning the period from 1749 to CE 2010 with a dating uncertainty of ±4% determined from repeated lamina-counting [27]. Due to some of the losses in preparing a thin section for magnetic scanning, the interval in this study is set as 1767–2006 CE.

### 2.2 Magnetic measurements

Magnetic scanning was conducted on stalagmite WY33 using a room-temperature Hall probe subjected to isothermal remanent magnetization (IRM) in pulsed fields of 100 mT at the State Key Laboratory of Marine Geology, Tongji University. This approach, based on the procedure outlined by Yi et al. [28], generates high-resolution maps of the magnetic field from the sample surface, revealing microscale magnetic signals [29,30]. The scanning system consists of a stationary axial Hall probe (∼100 µm sensor) mounted on an automated 2D motorized stage with a spatial resolution of 1 µm. Each measurement was repeated 30 times at intervals of 80 µm (approximately 3–5 points per year) along the same profile, with the Hall probe positioned < 0.5 mm from the sample surface. The final magnetic signal represents the average of the repeated scans, and two additional parallel tracks with ±1 mm offsets were acquired to verify signal reliability (also repeated 30 times).

The hysteresis loop, IRM acquisition curve, and first-order reversal curve (FORC) were measured using a Princeton Measurements Corporation MicroMag 3900 vibrating sample magnetometer (VSM), with a maximum field of 1 T. The IRM data was decomposed into coercivity components using the MAX UnMix application [31]. FORC diagrams were generated using the VARIFORC protocol [32] through the FORCinel software [33].

To further identify magnetic minerals in stalagmite WY33, magnetic extractions of a soil sample from the stalagmite surface were analyzed using a scanning electron microscope (SEM), following the method of Xue et al. [34]. SEM experiments were carried out on a LEO1450VP SEM equipped with an INCA ENERGY 300 Energy Dispersive X-ray Spectrometer (EDS) system at the Electron Microscopy Lab in the Institute of Geology and Geophysics, Chinese Academy of Sciences.

### 2.3 Calculating the seasonality index

Several indices have been developed to quantify precipitation seasonality [14; and references therein], and a higher proxy value indicates a higher degree of seasonal variation in rainfall, while a lower proxy value represents a more even distribution pattern in a year. In this study, the meteorological rainfall data of 740 stations across China, including Wudu, and the CN05.1 dataset were both collected from the China Meteorological Administration. The seasonality index was simply calculated as the standard deviation of rainfall across the four seasons:

(1) $$Seasonality=\;\sqrt{\frac{\sum {\left(x_{i}-\mu \right)}^{2}}{4}}$$

where *x_i_* represents the seasonal rainfall, and *µ* denotes the mean value of four seasons within a year. The seasonality index derived from IRM records (Seasonality_IRM_) was calculated using IRM values corresponding to 4 ± 2 points per year. In total, three seasonality indices were obtained: Seasonality_WD_ (based on Wudu station data), Seasonality_CN05_ (based on the CN05.1 dataset), and Seasonality_IRM_ (based on IRM data). Another index based on a different formula using an earlier version of the CN05.1 dataset for mainland China [14] was used as a reference (denoted as Seasonality_MAO_).

### 2.4 Timeseries forecasting

A convolutional neural network (CNN)-based time series forecasting model [35] was developed to analyze long-term observational data, following the procedure of Li et al. [36]. A sliding window approach was adopted to enable the model to leverage the preceding data to predict the subsequent year. To ensure comparability across variables with differing scales, all input data were normalized through linear transformation to a standardized range between 0 and 1. This normalization involved adjusting the minimum and maximum values of each variable, ensuring that all features contributed proportionately during the model’s training process. Following the generation of predictions, the outputs were re-transformed to their original scale to facilitate accurate interpretation of the results.

The model was trained with the Adam optimizer [35,36]. The initial learning rate was set to 1e-3 and was reduced by a factor of 0.1 after 400 epochs to stabilize convergence. To further enhance the model’s generalization capability and mitigate overfitting, L2 regularization was applied with a coefficient of 1e-3. Upon completing the training phase, the model was employed to generate future forecasts for the period 2007 to CE 2050 using a recursive forecasting method. In each iteration, the model was updated with the latest 80 years of data, ensuring that subsequent predictions were based on the most recent trends and patterns, thereby enhancing predictive accuracy.

Two parameters were employed to evaluate the forecasting model, namely the root mean squared error (RMSE) and the mean absolute error (MAE), as broadly used in previous studies [[37], [38], [39]]. RMSE serves as a core accuracy metric for prediction models, quantifying the difference between predicted and actual values. MAE calculates the average of the prediction errors, which simply averages the absolute difference between the predicted and actual values. Smaller RMSE and MAE values demonstrate the robustness of the model [37,40,41]. In this study, considering the balance between prediction errors and computability [42], RMSE and MAE were both < 0.5 in our training processes.

## 3 Results and analysis

### 3.1 Magnetic minerals in stalagmite WY33

Following paramagnetic correction, the hysteresis loops of one soil sample attached to the WY33 surface and one stalagmite subsample generally close at ∼0.3 T (Fig. 2), with no significant difference between the samples, indicating similar magnetic mineralogy in both the carbonate and soil samples. However, while the IRM acquisition curve of the soil sample is saturated above ∼0.5 T, indicating the presence of high-coercivity minerals, this high-coercivity component is not evident in the carbonate sample (Fig. S1). Decomposition of the IRM acquisition curve for the soil sample reveal four distinct coercivity components, with mode values of 12.9 mT, 51.7 mT, 125 mT, and 598 mT (Fig. 2f), confirming the presence of high-coercivity minerals.

**Fig. 2:**
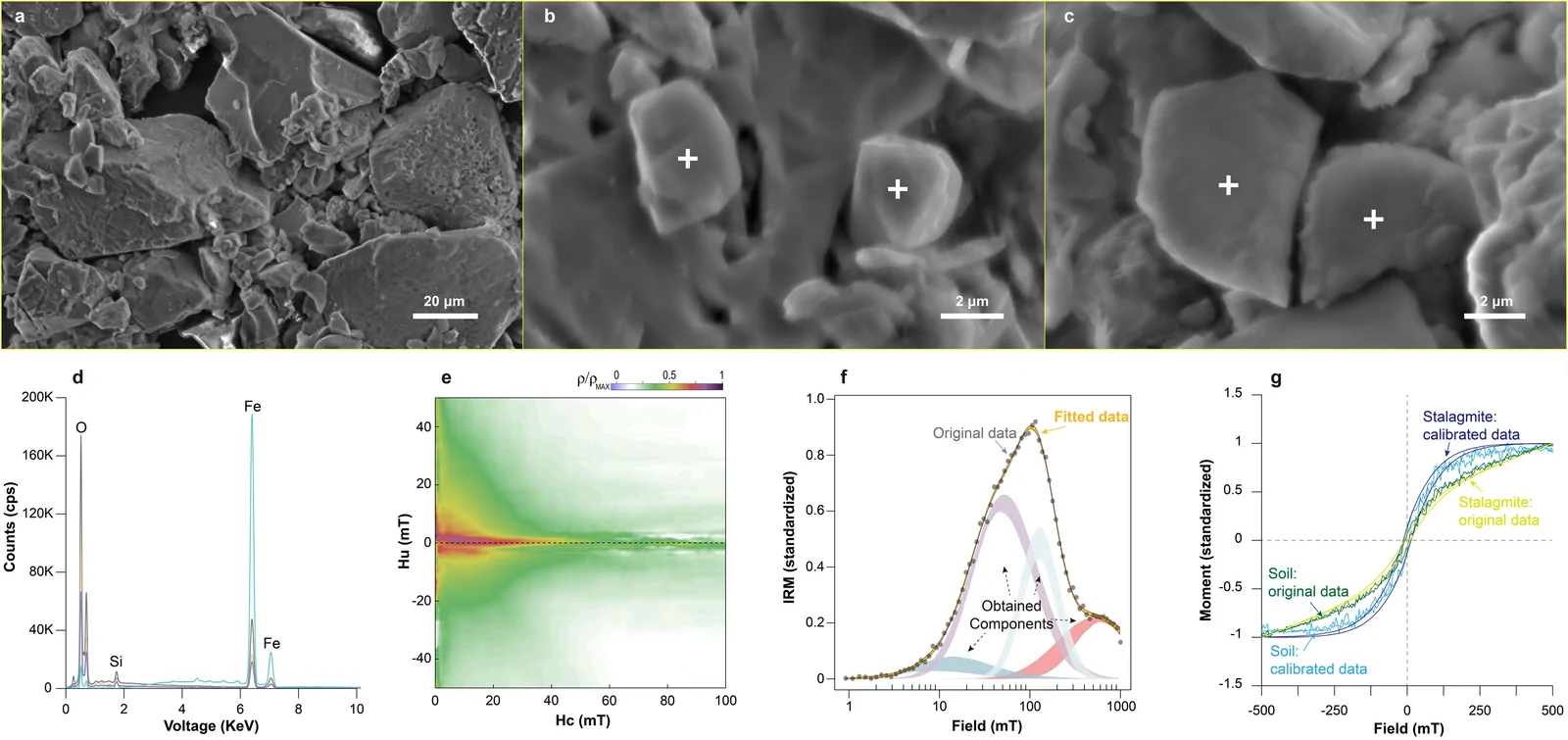
**Magnetic properties of magnetic minerals in the studied sample.** a-d, SEM and EDX analyses of magnetic minerals; e, FORC diagram; f, the unmixing result of IRM acquisition curve; g, hysteresis loops. There were two samples collected for analysis, including one soil sample attached to the surface of WY33 and one carbonate powder sample collected from the middle position of WY33 (∼40 mm from the top). Results shown in (a-f) were for the soil sample, and magnetic signals were not successfully detected in the carbonate powder sample due to few magnetic minerals.

The FORC diagram of the soil sample shows a wide vertical spreading below ∼40 mT (Fig. 2e), suggesting a mixing of multi-domain (MD) and pseudo-single domain (PSD) grains. Magnetic particles extracted from this soil sample are primarily micron-scale detrital magnetite with variable grain sizes, as observed through morphological characteristics (Fig. 2a-c) and EDX analysis (Fig. 2d), further confirming the bulk magnetic properties of the soil sample.

Previous studies suggested that the magnetic minerals found in stalagmites are primarily sourced from the surrounding environment, particularly soil-derived detrital and/or pedogenic magnetite [[43], [44], [45]]. It is proposed that increased rainfall can either enhance the production of pedogenic magnetite within the soil or increase the transport of detrital magnetic particles into the cave, both of which would lead to a higher concentration of magnetic minerals in cave deposits [46,47]. Given the magnetic properties of stalagmite WY33, it is inferred that the magnetite in WY33 likely originated from the soil and was incorporated into the stalagmite during its formation. In addition, magnetic minerals in the soil sample might have a more complex origination, possibly resulting in a difference in high-coercivity minerals and requiring further investigation in the future.

### 3.2 Scanning results

Due to the typically low concentration of magnetic minerals in speleothems compared to other detrital deposits, the natural magnetic signals of stalagmite WY33 are too weak to be detected using a stationary Hall probe. To enhance the detection of magnetic signals, the sample has been first subjected to an IRM field of 100 mT, which significantly amplifies the recorded signals. As a result, the magnetic scanning of stalagmite WY33 reveals a quasi-symmetrical pattern of fluctuation (Fig. 3a). This magnetic pattern is replicated along two additional measuring lines (Fig. S2), validating the robustness of the results.

**Fig. 3:**
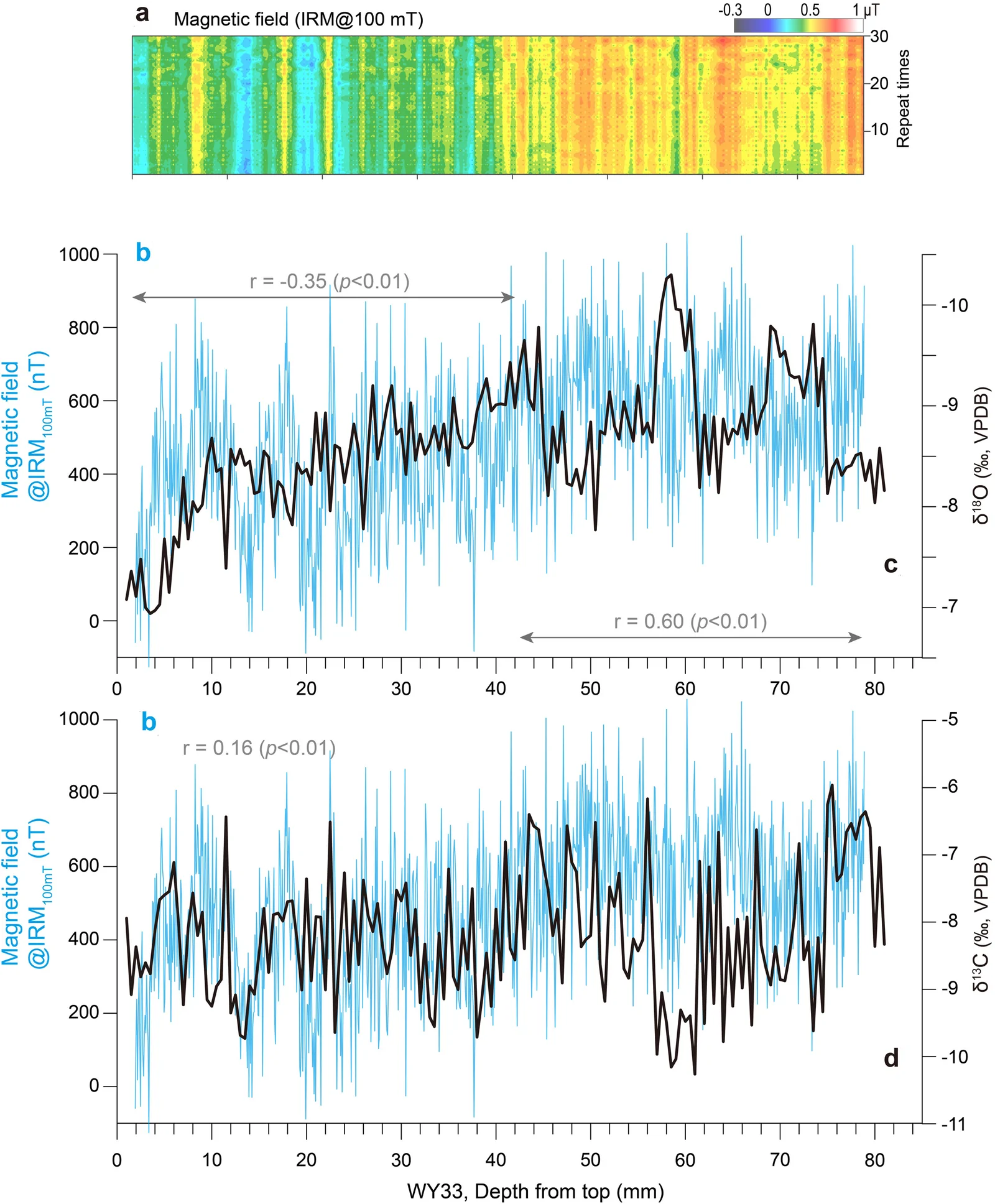
**Magnetic scanning of WY33 and comparison with isotopic records.** a, Magnetic scan along the major measuring line. b, The obtained IRM signal of stalagmite WY33. c-d, The published δ^18^O and δ^13^C records of stalagmite WY33 [27]. The correlation coefficients were calculated based on the data interpolated into annual resolution and presented as a 5-point moving average. The correlation coefficient between δ^18^O and δ^13^C records is 0.39 (*p* < 0.01).

Principal component analysis (PCA) is then applied to the magnetic scanning data to further interpret the signals. The leading component accounts for 85.6% of the total variance along the primary line, with similar outputs obtained along the two reference lines (83.4% and 76.3%, respectively). Given the consistency among the three scanning results (Fig. S3), the leading component is used to represent IRM changes in stalagmite WY33.

### 3.3 Significance of magnetic proxy

Previous studies have shown that magnetic minerals in stalagmites are positively correlated with rainfall changes [43,44,[46], [47], [48]], where higher magnetization values indicate greater rainfall. However, our comparison between IRM changes and δ^18^O and δ^13^C records from stalagmite WY33 suggests a more complex relationship. For example, no significant correlation is found between IRM and δ^18^O records over the entire period (Figs. 3 and S4), although two distinct subgroups are identified: a positive correlation before the 1880s (*r* = 0.60, *p* < 0.01) and a negative correlation after the 1880s (*r* = −0.35, *p* < 0.01).

The lack of correlation between magnetic signals and δ^18^O changes has been reported in stalagmite studies and may be attributed to the competing effects of temperature and moisture in cave environments [43,44,48]. Distinct relationships are also observed between regional rainfall and δ^18^O changes in the Onça Cave in Brazil [44]. The varying correlations during different periods may be linked to the “amount effect” and limited extratropical moisture sources for a negative correlation [49,50], or to the kinetic isotope effects related to relative humidity and temperature changes for a positive correlation [51,52].

Furthermore, extremely high IRM values (significant at *p* < 0.01) are identified in 1771, 1816 (the “Year Without Summer” in global climate), 1835, 1843, and CE 1886 (Fig. S5a), corresponding to documented flood events in the Yellow River catchment [53,54]. Given that the δ^18^O record of stalagmite WY33 has been interpreted as an indicator of regional hydroclimatic conditions [27], the coincidence of these events with low δ^18^O values suggests that higher rainfall increases the transport of detrital particles, including magnetite, into the cave system, which also aligns with regional rainfall proxy δ^18^O for the Chinese Loess Plateau [25,55].

However, the comparison between magnetic and δ^13^C changes in stalagmite WY33 reveals an opposite relationship (Figs. 3 and S4). δ^13^C is generally considered a proxy for regional vegetation [56]. With lower δ^13^C values indicating wetter conditions and better vegetation cover, the correlation between magnetic and δ^13^C changes (*r* = 0.16, *p* < 0.01) suggests that during wetter periods, increased vegetation could reduce the transport of detrital particles into the cave [[56], [57], [58]], thus lowering IRM values.

Therefore, although the covariations of magnetic and δ^18^O and δ^13^C records may suggest complex relationships between magnetic properties and other proxies, the significance of these magnetic properties in reflecting regional precipitation can be confirmed, and further evidence is required to fully understand these differing relations.

### 3.4 IRM-derived seasonality

To address the inconsistencies in the relationship between magnetic properties and rainfall changes, we derive a precipitation seasonality proxy (Seasonality_IRM_) based on the intra-annual IRM variance, which is independent of correlation directions (Fig. 3). Higher Seasonality_IRM_ values indicate greater seasonal variation in rainfall, and *vice versa*. The Seasonality_IRM_ record exhibits significant fluctuations, characterized by quasiperiodic oscillations (Fig. S5b), without a long-term trend in the past three centuries (Fig. 4). The consistency between various seasonality (Fig. 4a-c), including those using the same formula (Seasonality_WD_ and Seasonality_CN05_) or those using similar datasets (Seasonality_CN05_ and Seasonality_MAO_), further highlights the robustness of our findings and the validity of the Seasonality_IRM_ record as a representative measure for mainland China. In addition, minor differences observed such as in the 1980s between various seasonality proxies, which is also evident in comparison between the WY33 δ^18^O record and the reconstructed runoff in the middle Yellow River [27], could be attributed to differences in data resolution, sampling paths, and the distinct processes governing mineral magnetism and isotopic fractionation [59].

**Fig. 4:**
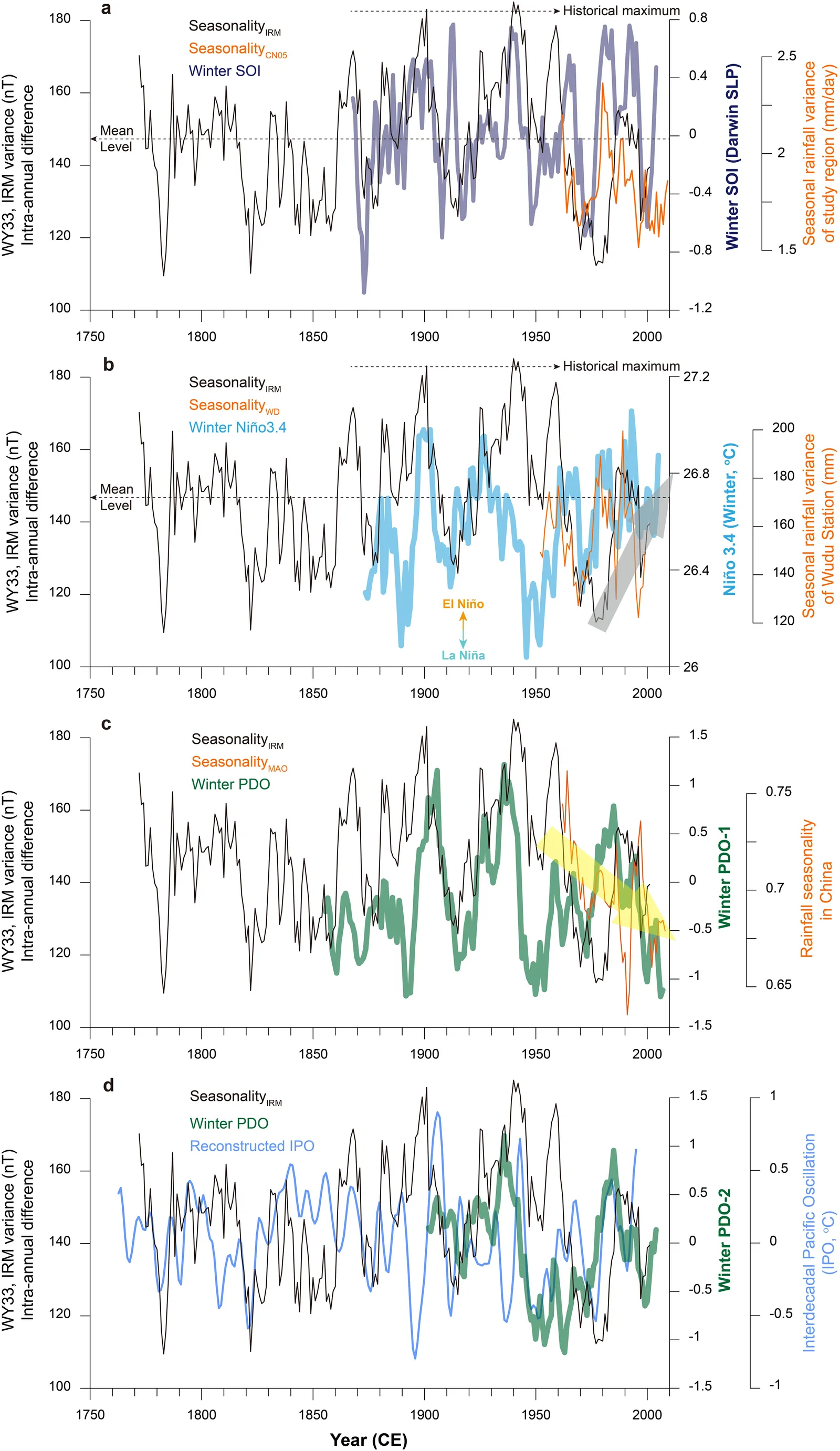
**Comparison between rainfall seasonality derived from magnetic scanning (this study) and various climatic proxies of the upper ocean.** a, Intra-annual IRM standard deviation, indicating regional precipitation seasonality (Seasonality_IRM_ derived in this study), *versus* the derived precipitation seasonality in the Chinese Loess Plateau [31°–35° N, 103°–107° E; 1961–2020 CE] from the CN05.1 dataset (Seasonality_CN05_), and winter (October-November-December, OND) condition of Southern Oscillation Index (SOI) [66,67]. SLP, sea level pressure. b, Seasonality_IRM_, *versus* the meteorological seasonality at Wudu station by calculating the standard deviation of four seasons’ rainfall (Seasonality_WD_), and the Niño 3.4 index during winters [66,67]. c, Seasonality_IRM_, *versus* the average seasonality in mainland China (Seasonality_MAO_) [14], and winter condition of PDO-1 [66,68]. Arrows in (b, c) indicate short-term trends of seasonality. d, Intra-annual IRM standard deviation (this study), *versus* winter PDO-2 from NOAA Extended Reconstructed SST V5 (ERSST) [26], which is a global monthly sea surface temperature dataset derived from the International Comprehensive Ocean-Atmosphere Dataset (ICOADS), and the reconstructed Interdecadal Pacific Oscillation (IPO) based on Sr/Ca ratio of Fiji corals [69]. IRM data were presented as 11-point moving averages, while the others were presented as 5-point moving averages. Spring (March-April-May, MAM) and winter (December-January-February, DJF) PDO, SOI, and Niño were also compared, and due to similar results, these comparisons were not shown here.

Moreover, decadal-scale events characterized by low regional rainfall seasonality are identified during 1782–1785, 1820–1829, 1842–1860, 1912–1915, and 1967–1983 CE. Conversely, periods with high precipitation seasonality are observed during 1866–1869, 1896–1901, 1935–1946, and 1956–1960 CE. Compared to rainfall reconstructions for the Chinese Loess Plateau based on tree-ring studies [60,61], these high-seasonality intervals are not solely associated with flood or drought events, but are characterized by frequent alternations between floods and droughts. Power spectrum analysis reveals that the most significant periodicities of the WY33 IRM series are 2–7 and 14 years (Fig. S6). The cycle of 2–7 years is associated with ENSO changes, while the 14-year cycle could be from ENSO, Pacific decadal oscillation (PDO), or North Atlantic Oscillation (NAO) conditions [[62], [63], [64], [65]], both of which suggest potential links to large-scale external forcing.

## 4 Discussion

### 4.1 Seasonality variability

Studies on precipitation seasonality in China have been largely limited to the modern meteorological period (1950s-2010s), resulting in discrepancies regarding observed trends. For example, analyses using similar rainfall datasets have yielded conflicting conclusions: Li et al. found no significant trend in precipitation seasonality in North China [18], whereas Sun et al. reported a decreasing trend since CE 1957 [19], which was later confirmed by Mao et al. [14]. Given the relatively short time span of modern observations, our IRM-derived reconstruction of precipitation seasonality provides valuable long-term context to assess variability and driving forces.

As shown in our analysis, both Seasonality_IRM_ and Seasonality_MAO_ proxies indicate a descending trend in precipitation seasonality over the past fifty years (Fig. 4c). However, this trend remains within the range of variability observed over the past century. Notably, no long-term trend is detected over the entire period (1767–2006 CE), yet the Seasonality_IRM_ record shows an ascending trend since the 1980s, suggesting that precipitation seasonality may be entering an upward phase (Fig. 5a), at least returning to an average historical level (Fig. 4b).

**Fig. 5:**
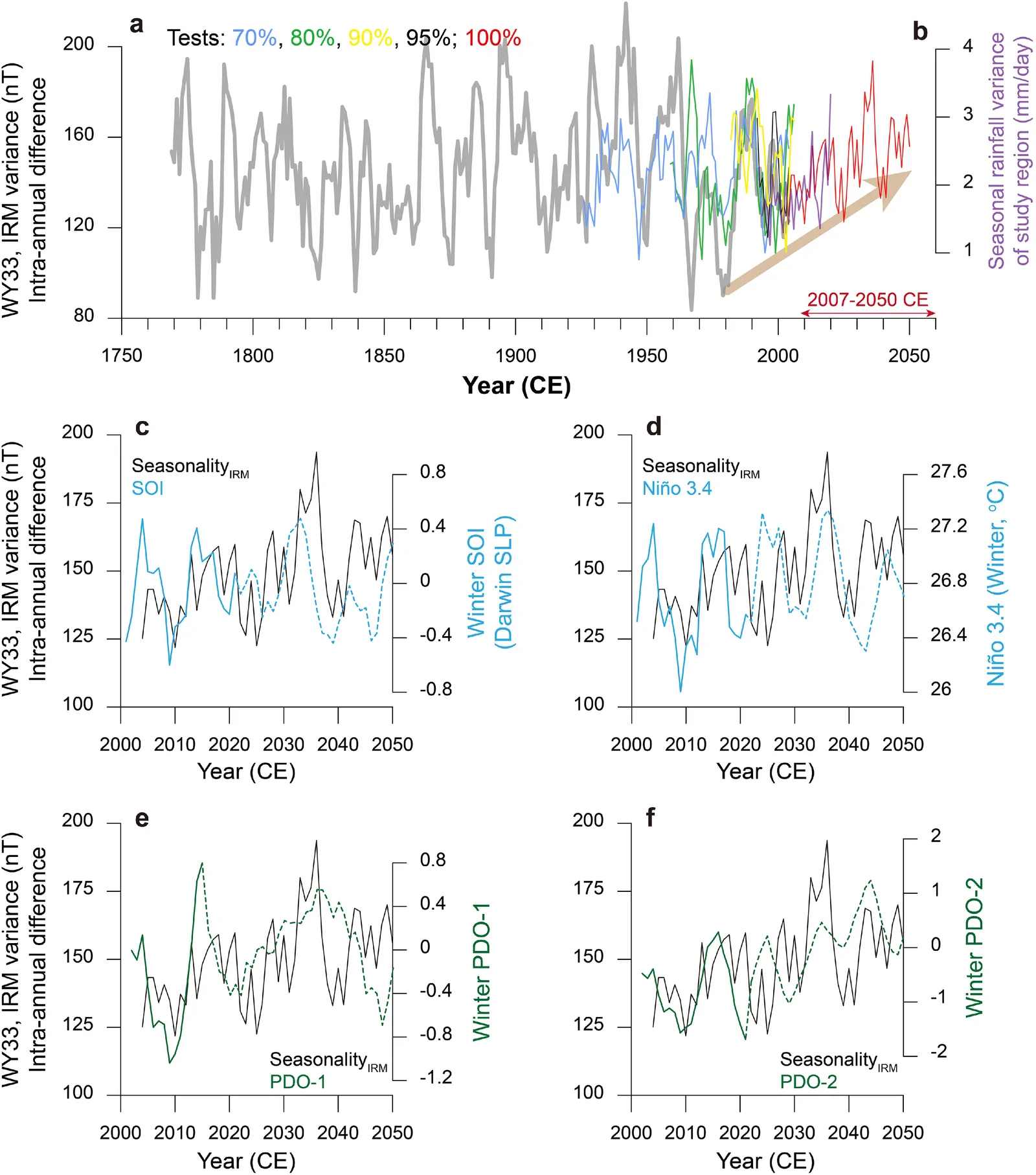
**Forecasting seasonality and its comparison to various climate variabilities.** (a) Seasonality_IRM_ derived in this study is presented as a 3-point moving average with the predicted records. “Tests” indicates 70%, 80%, 90%, 95%, and 100% of the total data were used for CNN training to forecast 30%, 20%, 10%, 5%, and 2007–2050 CE, respectively, representing the robustness of the prediction. (b) Seasonality_CN05_. (c-f) Comparisons between the predicted Seasonality_IRM_ and various proxies (solid lines, original data; dashed lines, predicted data following the same procedure for the Seasonality_IRM_). See notes in Fig. 4.

### 4.2 Regulating factors of seasonality

Our reconstruction demonstrates a close relationship between ENSO conditions and precipitation seasonality in North China. The variability observed in the Seasonality_IRM_ record aligns well with changes in the Southern Oscillation Index (SOI) and Niño 3.4 index (Fig. 4), and forecasting models predict that precipitation seasonality will continue to increase during 2007–2050 CE (Fig. 5c, d), which is consistent with some ENSO predictions in the coming decades [70]. Therefore, the predicted ascending trend observed in the seasonality and ENSO records may indicate a strong connection between the two. Spatial correlation analysis further indicates that precipitation seasonality in the Chinese Loess Plateau is tightly linked to sea surface temperature (SST) changes in the Pacific (Fig. 6). For example, a positive correlation is observed between seasonality and SST changes of the eastern Pacific in most of the months of the previous year. This spatial pattern indicates that higher SST in the eastern Pacific could lead to greater seasonality in North China, likely highlighting the dominant role of El Niño events in modulating the monthly/seasonal distribution of precipitation in the past three centuries and the coming decades [21,71,72].

**Fig. 6:**
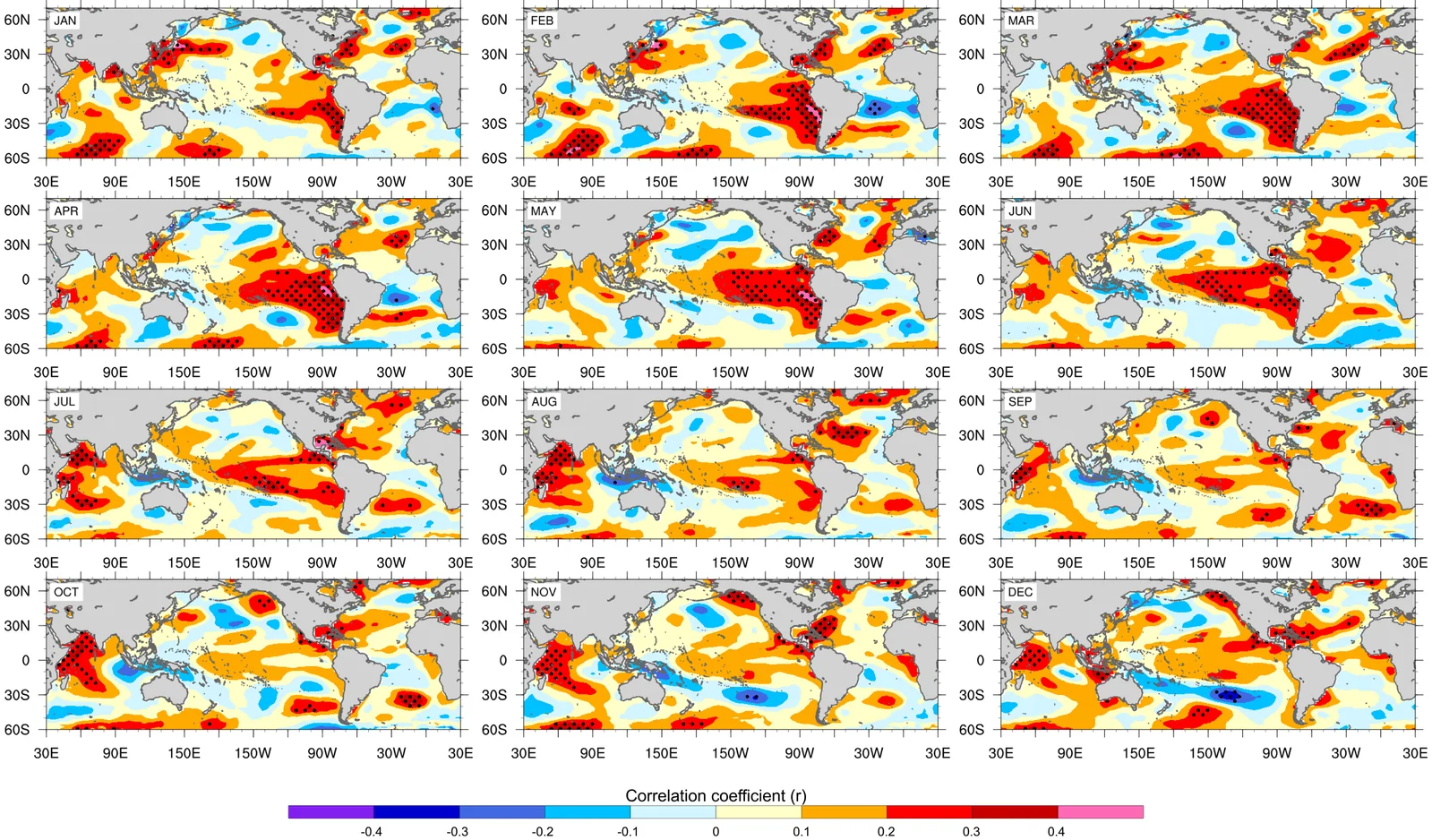
**Spatial correlation analysis of precipitation seasonality in the Chinese Loess Plateau and one-year leading SST changes in the Pacific**. The precipitation seasonality data [31°–35° N, 103°–107° E; 1961–2020 CE] was from the CN05.1 dataset (Seasonality_CN05_). CN05.1 dataset is constructed by the “anomaly approach” during the interpolation but with more station observations (∼2400) in China [77,78]. CN05.1 comprised four variables of daily mean, maximum and minimum temperature, and precipitation in original and was updated with the inclusion of daily mean humidity, wind speed, and surface evaporation [79]. The SST data in the Pacific were from ERSST [26]. Black dots represent the correlation coefficients being significant at *p* < 0.05 level.

However, it has been claimed that El Niño events would increase annual rainfall and extend wet seasons in North China, which would reduce the intra-annual difference in precipitation, thus leading to smaller seasonality [14]. This proposed teleconnection can be similarly observed in the spatial correlation without lagging/leading phases (Fig. S7), in which a positive correlation and a negative correlation were observed between seasonality and SST changes of the western Pacific and eastern Pacific, respectively, during the latter half of the year.

The conflicting observations in the preceding and simultaneous ENSO appear to be explained by the lagging effect of ENSO changes on precipitation in North China [73,74], which is also evident in the North Atlantic [75]. For example, the leading El Niño events have more robust influences on the anomalous summer rainfall in monsoonal regimes of China through distinct vapor transport and wind patterns [76], likely suggesting that simultaneous SST conditions in the eastern Pacific during the latter half of the year could not provide sufficient impacts on annual distribution of rainfall changes in the North China.

In addition to ENSO, other climate oscillations, such as the PDO, Interdecadal Pacific Oscillation (IPO), NAO, and Indian Ocean Dipole (IOD) also influence precipitation seasonality in China [80]. Our results show significant correlations between the Seasonality_IRM_ record and the PDO and IPO (Fig. 4c, 4d), similarly with a predicted ascending trend during 2007–2050 CE (Fig. 5e, f), as well as spatial correlations with SST anomalies in the North Pacific, North Atlantic, and West Indian Ocean (Fig. 6). These findings suggest that the PDO, IPO, NAO and IOD may exert secondary influences on seasonality by modulating moisture availability and upper-ocean conditions over longer timescales.

Overall, our IRM-based reconstruction highlights the importance of considering multiple climatic forcing, particularly ENSO and PDO, when assessing precipitation seasonality. These results emphasize the complexity of seasonal rainfall patterns in monsoonal regions and underscore the need for further research to disentangle the interactions between these oscillations and regional precipitation trends.

## 5 Concluding remarks

This study presents a high-resolution reconstruction of precipitation seasonality in the Chinese Loess Plateau, spanning the period from 1767 to CE 2006. By applying magnetic scanning to stalagmite WY33 and comparing the results with modern meteorological data, we have identified significant fluctuations in precipitation seasonality characterized by quasi-oscillations with periodicities of 2–7 and 14 years. These variations in seasonality are closely linked to ENSO conditions, with El Niño events amplifying precipitation seasonality in North China on decadal timescales. The observed upward trend in precipitation seasonality since the 1980s suggests that North China may be entering a new phase of increased rainfall seasonality, which could have significant implications for water-resource management policies, which are currently based on meteorological records. Increased precipitation seasonality may result in greater variability in water availability, potentially affecting agricultural practices [81,82] and ecosystem sustainability [83,84] and challenging existing strategies for managing water resource and ecosystem vulnerability [13]. Therefore, these findings underscore the complexity of seasonal rainfall dynamics in monsoonal regions and highlight the need for further research to better understand the interactions between multiple climate drivers and regional precipitation trends.

## Declaration of interest statement

The authors declare that they have no conflicts of interest in this work.

## CRediT authorship contribution statement

**Liang Yi:** Writing – original draft, Formal analysis, Data curation. **Liangcheng Tan:** Writing – review & editing, Investigation, Conceptualization.

## Appendix Supplementary materials

Image, application 1

## References

[1] Easterling D.R., Meehl G.A., Parmesan C. (2000). Climate extremes: Observations, modeling, and impacts. Science.

[2] Konapala G., Mishra A.K., Wada Y. (2020). Climate change will affect global water availability through compounding changes in seasonal precipitation and evaporation. Nat. Commun..

[3] Pendergrass A.G., Knutti R. (2018). The uneven nature of daily precipitation and its change. Geophys. Res. Lett..

[4] Dubois N., Oppo D.W., Galy V.V. (2014). Indonesian vegetation response to changes in rainfall seasonality over the past 25,000 years. Nat. Geosci..

[5] Piao S., Liu Q., Chen A. (2019). Plant phenology and global climate change: Current progresses and challenges. Global Change Biol..

[6] Piao S., Ciais P., Huang Y. (2010). The impacts of climate change on water resources and agriculture in China. Nature.

[7] Villarreal S., Vargas R., Yepez E.A. (2016). Contrasting precipitation seasonality influences evapotranspiration dynamics in water-limited shrublands. J. Geophys. Res.-Biogeo..

[8] Le P.V.V., Randerson J.T., Willett R. (2023). Climate-driven changes in the predictability of seasonal precipitation. Nat. Commun..

[9] Sahany S., Mishra S.K., Pathak R. (2018). Spatiotemporal variability of seasonality of rainfall over India. Geophys. Res. Lett..

[10] Berg P., Moseley C., Haerter J.O. (2013). Strong increase in convective precipitation in response to higher temperatures. Nat. Geosci..

[11] Fischer E.M., Knutti R. (2016). Observed heavy precipitation increase confirms theory and early models. Nat. Clim. Change.

[12] Kirchmeier-Young M.C., Zhang X. (2020). Human influence has intensified extreme precipitation in North America. P. Natl. A. Sci..

[13] Feng X., Porporato A., Rodriguez-Iturbe I. (2013). Changes in rainfall seasonality in the tropics. Nat. Clim. Change.

[14] Mao Y., Wu G., Xu G. (2022). Reduction in precipitation seasonality in China from 1960 to 2018. J. Climate.

[15] Wang S.Y.S., Yoon J.-H., Becker E. (2017). California from drought to deluge. Nat. Clim. Change.

[16] Gebrechorkos S.H., Hülsmann S., Bernhofer C. (2019). Long-term trends in rainfall and temperature using high-resolution climate datasets in East. Africa. Sci. Rep..

[17] Pryor S.C., Schoof J.T. (2008). Changes in the seasonality of precipitation over the contiguous USA. J. Geophys. Res.-Atmos..

[18] Li X., Jiang F., Li L. (2011). Spatial and temporal variability of precipitation concentration index, concentration degree and concentration period in Xinjiang, China. Int. J. Climatol..

[19] Sun Q., Miao C., Duan Q. (2017). Changes in the spatial heterogeneity and annual distribution of observed precipitation across China. J. Climate.

[20] Cai W., Santoso A., Collins M. (2021). Changing El Niño–Southern oscillation in a warming climate. Nat. Rev. Earth Env..

[21] Sun Q., Miao C., AghaKouchak A. (2020). Possible increased frequency of enso-related dry and wet conditions over some major watersheds in a warming climate. B. Am. Meteorol. Soc..

[22] Lv A., Qu B., Jia S. (2019). Influence of three phases of El Niño–Southern Oscillation on daily precipitation regimes in China. Hydrol. Earth Syst. Sci..

[23] Xu C., Wang S.Y.S., Borhara K. (2023). Asian-Australian summer monsoons linkage to ENSO strengthened by global warming. Npj Clim. Atmos. Sci..

[24] Yang Y., Piper D.J.W., Xu M. (2022). Northwestern Pacific tropical cyclone activity enhanced by increased Asian dust emissions during the little ice age. Nat. Commun..

[25] Tan L., Li Y., Wang X. (2020). Edwards, holocene monsoon change and abrupt events on the western Chinese loess plateau as revealed by accurately dated stalagmites. Geophys. Res. Lett..

[26] B. Huang, P.W. Thorne, V.F. Banzon, et al., NOAA Extended Reconstructed Sea Surface Temperature (ERSST), Version 5, N.N.C.f.E. Information, 2017, doi:10.7289/V5T72FNM.

[27] Tan L., An Z., Huh C.-A. (2014). Cyclic precipitation variation on the western Loess Plateau of China during the past four centuries. Sci. Rep..

[28] Yi L., Medina-Elizalde M., Kletetschka G. (2020). The potential of marine ferromanganese nodules from eastern pacific as recorders of earth’s magnetic field changes during the past 4.7 myr: A geochronological study by magnetic scanning and authigenic ^10^Be/^9^Be dating. J. Geophys. Res.-Sol. Ea..

[29] Cheng Y., Xu Y., Yi L. (2023). Chronology and critical metals enrichment mechanism of ferromanganese nodules from the Parece Vela Basin, Philippine Sea. Chem. Geol..

[30] Yi L., Li Y., Mikhailik P. (2023). Magnetic and geochemical scanning reveals growth history of marine ferromanganese crust on Detroit Seamount, northwest Pacific since the early miocene. Quatern. Int..

[31] Maxbauer D.P., Feinberg J.M., Fox D.L. (2016). MAX UnMix: A web application for unmixing magnetic coercivity distributions. Comput. Geosci..

[32] Egli R. (2013). VARIFORC: An optimized protocol for calculating non-regular first-order reversal curve (FORC) diagrams. Global Planet Change.

[33] Harrison R.J., Feinberg J.M. (2008). FORCinel: An improved algorithm for calculating first-order reversal curve distributions using locally weighted regression smoothing. Geochem. Geophy. Geosy..

[34] Xue P., Chang L., Thomas E. (2023). Abrupt Northwest Atlantic deep-sea oxygenation decline preceded the palaeocene-eocene thermal maximum. Earth Planet. *Sc*. Lett..

[35] Gu J., Wang Z., Kuen J. (2018). Recent advances in convolutional neural networks. Pattern Recogn.

[36] Li Z., Liu F., Yang W. (2022). A survey of convolutional neural networks: Analysis, applications, and prospects. IEEE T. Neur. Net. Lear..

[37] Hodson T.O., Over T.M., Foks S.S. (2021). Mean squared error, deconstructed. J. Adv. Model. Earth Sy..

[38] Karunasingha D.S.K. (2022). Root mean square error or mean absolute error? Use their ratio as well. Inform. Sciences.

[39] Kaur G., Saini S. (2024). Comparative analysis of RMSE and MAP metrics for evaluating CNN and LSTM models. AIP Conf. Proc..

[40] Das K., Jiang J., Rao J.N.K. (2004). Mean squared error of empirical predictor. Ann. Statist..

[41] Yuan S. (2022). Review of root-mean-square error calculation methods for large deployable mesh reflectors. Int. J. Aerospace Eng..

[42] Chai T., Draxler R.R. (2014). Root mean square error (RMSE) or mean absolute error (MAE)? – Arguments against avoiding RMSE in the literature. Geosci. Model Dev..

[43] Zhu Z., Feinberg J.M., Xie S. (2017). Holocene ENSO-related cyclic storms recorded by magnetic minerals in speleothems of central China. P. Natl. A. Sci..

[44] Hess K., Fu R.R., Piascik S. (2024). Investigating speleothem magnetism as a proxy for dust mobilization and rainfall. Quaternary Sci. Rev..

[45] Denniston R.F., Luetscher M. (2017). Speleothems as high-resolution paleoflood archives. Quaternary Sci. Rev..

[46] Bourne M.D., Feinberg J.M., Strauss B.E. (2015). Long-term changes in precipitation recorded by magnetic minerals in speleothems. Geology.

[47] Xie S., Evershed R.P., Huang X. (2013). Concordant monsoon-driven postglacial hydrological changes in peat and stalagmite records and their impacts on prehistoric cultures in central China. Geology.

[48] Chen Q., Zhang T.W., Wang Y.T. (2019). Magnetism signals in a stalagmite from southern China and reconstruction of paleorainfall during the interglacial-glacial transition. Geophys. Res. Lett..

[49] Dansgaard W. (1964). Stable isotopes in precipitation. Tellus.

[50] Cruz F.W., Karmann I., Viana O. (2005). Stable isotope study of cave percolation waters in subtropical Brazil: Implications for paleoclimate inferences from speleothems. Chem. Geol..

[51] Riechelmann D.F.C., Deininger M., Scholz D. (2013). Disequilibrium carbon and oxygen isotope fractionation in recent cave calcite: Comparison of cave precipitates and model data. Geochim. Cosmochim. Ac..

[52] Stríkis N.M., Buarque P.F.S.M., Cruz F.W. (2024). Modern anthropogenic drought in Central Brazil unprecedented during last 700 years. Nat. Commun..

[53] Hu M., Luo C. (1992).

[54] Zhang D. (2013).

[55] Chen L., Wang T., Sinha A. (2024). A seasonally resolved stalagmite δ^18^O record indicates the regional activity of tropical cyclones in Southeast China. Npj Clim. Atmos. Sci..

[56] Baker A., Ito E., Smart P.L. (1997). Elevated and variable values of ^13^C in speleothems in a British cave system. Chem. Geol..

[57] McDermott F. (2004). Palaeo-climate reconstruction from stable isotope variations in speleothems: A review. Quaternary Sci. Rev..

[58] Tremaine D.M., Froelich P.N., Wang Y. (2011). Speleothem calcite farmed in situ: Modern calibration of δ^18^O and δ^13^C paleoclimate proxies in a continuously-monitored natural cave system. Geochim. Cosmochim. Ac..

[59] Jaqueto P., Trindade R.I.F., Hartmann G.A. (2016). Linking speleothem and soil magnetism in the Pau d'Alho cave (central South America). J. Geophys. Res.-Sol. Ea..

[60] Liu Y., Sun B., Song H. (2013). Tree-ring-based precipitation reconstruction for Mt. Xinglong, China, since AD 1679. Quatern. Int..

[61] Yi L., Yu H., Ge J. (2012). Reconstructions of annual summer precipitation and temperature in north-central China since 1470 AD based on drought/flood index and tree-ring records. Clim. Chang..

[62] Bruun J.T., Allen J.I., Smyth T.J. (2017). Heartbeat of the southern oscillation explains ENSO climatic resonances. J. Geophys. Res.-Oceans.

[63] Kwon Y.-O., Riser S.C. (2004). North Atlantic subtropical mode water: A history of ocean-atmosphere interaction 1961–2000. Geophys. Res. Lett..

[64] Zhao Y., Zou X., Cao L. (2014). Changes in precipitation extremes over the pearl river basin, southern China, during 1960–2012. Quatern. Int..

[65] Labat D. (2010). Cross wavelet analyses of annual continental freshwater discharge and selected climate indices. J. Hydrol..

[66] Schneider D.P., Deser C., Fasullo J. (2013). Climate data guide spurs discovery and understanding. Eos, Trans. Amer. Geophys. Union.

[67] Trenberth K., Staff N.C.f.A.R. (2024). The Climate Data Guide: Southern Oscillation Indices: Signal, Noise and Tahiti/Darwin SLP (SOI).

[68] Deser C., Trenberth K., Staff N.C.f.A.R. (2022). The Climate Data Guide: Pacific Decadal Oscillation (PDO): Definition and Indices.

[69] D’Olivo J.P., Zinke J., Goyal R. (2024). Coral Sr/Ca-SST reconstruction from Fiji extending to ∼1370 CE reveals insights into the Interdecadal Pacific Oscillation. Sci. Adv..

[70] Geng T., Cai W., Jia F. (2024). Decreased ENSO post-2100 in response to formation of a permanent El Niño-like state under greenhouse warming. Nat. Commun..

[71] Ma F., Ye A., You J. (2018). 2015–16 floods and droughts in China, and its response to the strong El Niño. Sci. Total Environ..

[72] Lu B., Li H., Wu J. (2019). Impact of El Niño and Southern Oscillation on the summer precipitation over Northwest China. Atmos. Sci. Lett..

[73] Ailikun B., Yasunari T. (2001). ENSO and asian summer monsoon: Persistence and transitivity in the seasonal March. J. Meteorol. Soc. Jan.. Ser. II.

[74] Sun L., Yang X.-Q., Tao L. (2021). Changing impact of ENSO events on the following summer rainfall in eastern china since the 1950s. J. Clim..

[75] Scaife A.A., Dunstone N., Hardiman S. (2024). ENSO affects the North Atlantic Oscillation 1 year later. Science.

[76] Zhang P., Xie S.-P., Kosaka Y. (2024). Why East Asian monsoon anomalies are more robust in post El Niño than in post La Niña summers. Nat. Commun..

[77] Wu J., Gao X. (2013). A gridded daily observation dataset over China region and comparison with the other datasets. Chinese J. Geophys..

[78] Xu Y., Gao X., Shen Y. (2009). A daily temperature dataset over China and its application in validating a RCM simulation. Adv. Atmos. Sci..

[79] Wu J., Gao X., Giorgi F. (2017). Changes of effective temperature and cold/hot days in late decades over China based on a high resolution gridded observation dataset. Int. J. Climatol..

[80] Deng S., Yang N., Li M. (2019). Rainfall seasonality changes and its possible teleconnections with global climate events in China. Clim. Dynam..

[81] Fang H., Sha M., Xie Y. (2023). Shifted global vegetation phenology in response to climate changes and its feedback on vegetation carbon uptake. Remote Sens.-Basel.

[82] Cleland E.E., Chuine I., Menzel A. (2007). Shifting plant phenology in response to global change. Trends Ecol. Evol..

[83] Walther G.-R., Post E., Convey P. (2002). Ecological responses to recent climate change. Nature.

[84] Parmesan C. (2006). Ecological and evolutionary responses to recent climate change. Annu. Rev. Ecol. Evol. S..

