# Supplementary material for: ENSO-induced precipitation seasonality in the Chinese loess plateau during 1767–2050 CE
Source: Fundam Res. 2025 Apr 23;6(5):3104–13. doi: 10.1016/j.fmre.2025.04.010 (PMC13644506; doi:10.1016/j.fmre.2025.04.010)
Supplement: Supplementary file 1 [file mmc1.docx]

### Supplementary Figures

**Fig.S1.** IRM acquisition curve of the two studied samples (stalagmite and soil).

**Fig. S2.** All magnetic scans of stalagmite WY33. The color scale is the same as shown in Fig. 3a.

**Fig. S3.** Correlation analysis between the three magnetic scans. Also see comparison in Fig. S2.

**Fig. S4.** Comparison between IRM changes and δ^18^O and δ^13^C records. All data were interpolated into annual resolution and presented as 5-point moving average. The correlation coefficient between δ^18^O and δ^13^C records is 0.39 (*p*<0.01).

**Fig. S5.** Two magnetic records derived from magnetic scanning of stalagmite WY33. Bold lines represent 5-point moving averages. The labelled years are the flooding events in history.

**Fig. S6.** Spectral analysis of the two IRM records based on the multi-taper method (MTM). The MTM code was from ref. [85,86]. CL80 and CL90, indicate a periodicity is significant at *p*<0.2 and 0.1 level, respectively.

**Fig. S7.** Spatial correlation analysis of precipitation seasonality and present-year SST changes in the Pacific. See notes in Fig. 6.

### Supplementary References

[85] M. Ghil, M.R. Allen, M.D. Dettinger, K. Ide, D. Kondrashov, M.E. Mann, A.W. Robertson, A. Saunders, Y. Tian, F. Varadi, and P. Yiou, Advanced spectral methods for climatic time series. Reviews of Geophysics 40 (2002) 3-1-3-41.

[86] M.D. Dettinger, M. Ghil, C.M. Strong, W. Weibel, and P. Yiou, Software expedites singular-spectrum analysis of noisy time series. Eos, Transactions American Geophysical Union 76 (1995) 12-21.
